# Accelerated whole-body protein catabolism in subjects with type 2 Diabetes Mellitus and albuminuria

**DOI:** 10.1371/journal.pone.0243638

**Published:** 2020-12-17

**Authors:** Michela Zanetti, Rocco Barazzoni, Edward Kiwanuka, Monica Vettore, Monica Vedovato, Paolo Tessari

**Affiliations:** 1 Metabolism Division, Department of Medicine, University of Padova, Padova, Italy; 2 DSM, University of Trieste, Trieste, Italy; Baker IDI Heart and Diabetes Institute, AUSTRALIA

## Abstract

**Background:**

Albuminuria develops in ~40% of subjects with Type 2 Diabetes Mellitus (T2DM), and is often associated with malnutrition, severe comorbidities and decreased life expectancy. The association between albuminuria and altered whole body protein turnover in T2DM is currently unknown.

**Objective:**

To assess whole body protein degradation and synthesis in type 2 diabetes with and without albuminuria.

**Methods:**

Fourteen T2DM male subjects, with either increased [AER+] or normal [AER-] urinary albumin excretion rate, and eleven age-matched male healthy controls, were infused with phenylalanine [Phe] and tyrosine [Tyr] tracers. Post-absorptive rates of appearance (Ra) of Phe (= protein degradation) and Tyr, Phe hydroxylation to Tyr (Hy) (catabolic pathway), and Phe disposal to protein synthesis [PS], were determined.

**Results:**

Phe and Tyr Ra were not different among the groups. However, in T2DM [AER+], the fraction of Phe disposal to hydroxylation was ~50% and ~25% greater than that of both controls and T2DM [AER-] (p<0.006 and p = 0.17, respectively). Conversely, as compared to controls, the fractional Phe disposal to PS was ~10% lower in T2DM [AER+] (p<0.006), and not different from that in T2DM [AER-]. As a consequence, in T2DM [AER+], the ratio between the fractional Phe disposal to hydroxylation and that to PS was ~70% greater (p = 0.005) than that in healthy controls, whereas in the T2DM [AER-] this ratio was ~30% greater than in controls (p = 0.19).

**Conclusions:**

On the basis of the kinetics of the essential amino acid phenylalanine, T2DM subjects with increased AER exhibit a catabolic pattern of whole body protein turnover.

## Introduction

Albuminuria (either micro- or macro-) may develop in as much as 40% of subjects with type 2 diabetes (T2DM) over their lifetime, and it is associated with chronic renal failure and/or end stage renal disease [1]. Albuminuria on turn strongly predicts adverse clinical outcomes and mortality in T2DM [2, 3]. Furthermore, albuminuric T2DM patients often exhibit a cluster of metabolic and nutritional abnormalities linked both to the metabolic disease and to kidney failure, including insulin resistance [4], cardiovascular comorbidities [5], poor nutritional status [6], decreased lean body mass [7] and sarcopenia [8]. Albuminuric T2DM patients may also exhibit hypoalbuminemia and decreased/altered concentrations and turnover of plasma proteins [9, 10]. Several causes can determine malnutrition, sarcopenia and protein-energy wasting in T2DM subjects with nephropathy. Decreased food intake, restriction of dietary proteins, limited physical activity, concurrent diseases, chronic therapy, can all play a role. Conversely, from a mechanistic standpoint, protein catabolism can be due either to increased proteolysis or decreased protein synthesis, or both. Detection of alterations at any of these steps may be important to adopt adequate strategies for prevention.

The essential amino acid leucine is an established marker of body protein turnover and catabolism. Although it has been previously reported that T2DM patients with albuminuria have normal whole-body leucine flux [9–11], suggesting no alterations of whole-body protein turnover, the rate(s) of irreversible catabolism of any essential amino acid, in respect to the “albuminuric” status, has not been specifically investigated in T2DM. This information is particularly relevant when addressing the therapeutic strategies to correct the nutritional abnormalities associated with renal disease.

Therefore, this study was designed to measure the kinetics of the essential amino acid phenylalanine, another well-accepted marker of whole body protein turnover and catabolism [12–14], in T2DM subjects with and without albuminuria, as well as in healthy control subjects. Both the catabolic and the anabolic route of phenylalanine disposal were determined in post-absorptive conditions.

## Methods

### Subjects

Fourteen male type 2 diabetic subjects (diabetes duration: >2.5 years), either without [AER-] or with [AER+] increased albumin excretion rate, were sequentially recruited from patients attending the Diabetes Centre at the University Hospital of Padova, Italy, for participation into a larger project aimed at investigating the effect of albuminuria on whole body and plasma protein kinetics. Inclusion criteria were: male gender (females were intentionally excluded to minimize sex-related confounding factors); age: between 40 and 70 years; absence of advanced systemic diseases besides diabetes, as well as of any anamnestic, clinical and laboratory sign of either glomerulonephritis, renal stone disease, major organ insufficiency, or clinically-overt inflammation. All subjects were in good general conditions. Background retinopathy and non-critical peripheral vascular stenosis were found in one subject of the T2DM [AER-] group. In the T2DM [AER+] subjects, four subjects had retinopathy (one background, three proliferative) and four peripheral vascular disease. No [AER-] T2DM subject was a current smoker or had being smoking for at least six months prior to the study, whereas in the [AER+] T2DM group two subjects were current smokers. No patient had clinical signs of either edema or pleural and abdominal liquid effusion. In T2DM subjects, the treatment of diabetes consisted in diet only (n = 1), diet plus oral hypoglycaemic agents (OHA) (sulphonylurea and/or biguanide, n = 2), suphonylurea and insulin (n = 1), or insulin (n = 3). In the [AER-] T2DM subjects, the hypoglycemic therapy consisted in diet only (n = 2) or in diet plus OHA (n = 5). In the [AER+] T2DM group, five patients were also treated for hypertension and one with fibrates, whereas in the [AER-] T2DM group five subjects were treated for hypertension. All drugs were suspended the evening before the study. The patients who agreed to participate to the study were sequentially enrolled. As controls, eleven male non diabetic, healthy subjects, matched by age to the diabetic groups, were enrolled and included into the study. Measurement of albuminuria was based on at least two 24-hr urine collections.

All subjects had been adapted to a standard weight-maintaining diet containing ~50% of calories as carbohydrates, ~20% as proteins and ~30% as lipids for at least two months. Dietary intake was carefully monitored by an expert dietician at the Diabetic Centre. Daily protein intake was unrestricted and estimated to be at least >0.8 grams/Kg of body weight in both patient groups. The protocol was approved by the Ethics Committee of the Medical Faculty at the University of Padova (approval number 686 AG/vb, Feb 9^th^, 1996), and it complied with the Helsinki Declaration. The aims and the potential risks of the study were explained in detail, and an informed consent was signed by each subject. The studies were initiated in 1998 and completed in 2013.

### Isotopes

L-[ring-^2^H_5_]-phenylalanine (D_5_-Phe) and L-[^2^H_2_]-tyrosine (D_2_-Tyr) were purchased from Masstrace (Woburn, MA). L-[ring-^2^H_4_]-tyrosine (D_4_-Tyr) was purchased from Eurisotop (Gif-Sur-Yvette, France). The stable isotope enrichments were >99% mole percent. All tracers were dissolved in sterile saline and proven to be sterile and pyrogen free before use.

### Experimental design

All subjects were admitted at the Clinical Study Unit on the morning of the study after the overnight fast. Continuous infusion of D_5_-Phe (at 0.045–0.047 μmol x kg^-1^ x min^-1^) and of D_4_-tyrosine (at 0.022–0.029 μmol x kg^-1^ x min^-1^) were started at ~07:30 A.M. by means of calibrated pumps. Priming doses of the isotopes (in the amount of 60 times the constant infusion rate per minute), as well as a priming dose of D_4_-Tyr (0.08 mg/kg), were also administered as boluses at the start of tracer infusions, and continued for 180 min. Venous-arterialized blood samples were drawn every 60' in the first two hours, and every 20’ between 120’ and 180’, at ~steady state of isotope and substrate concentrations.

### Biochemical determinations, calculations, and statistical analysis

Blood samples (10–12 ml) were collected into EDTA (6% w/v)-containing tubes, rapidly centrifuged, and the plasma was stored at -20° until assay. Plasma glucose, triglyceride, total and HDL cholesterol, creatinine, insulin, as well as urinary albumin concentrations, were determined by standard laboratory methods as reported elsewhere [9, 10]. The glomerular filtration rate (GFR) was estimated by the MDRD equation [15]. Plasma amino acid concentrations were measured with a Beckman Amino Acid Analyzer (Beckman Instruments, Palo Alto, CA, USA).

Plasma D_5_-Phe, D_2_-Tyr, and D_4_-Tyr mole percent enrichments were determined by GC-MS as tert-butyl-dimethyl-silyl derivatives and electron impact ionization [16]. The mass-to-charge ratios of monitored fragments were 239/234 for D_5_-Phe, 468/466 for D_2_-Tyr, and 470/466 for D_4_-Tyr. Enrichments were expressed as tracer-to-tracee ratios (TTR) [17].

The kinetic data were expressed as μmol x kg LBM^−1^ x min^−1^. Whole body phenylalanine and tyrosine rate of appearance (Ra) were calculated by standard steady–state formulas using plasma TTR [17].

The rate of phenylalanine hydroxylation to tyrosine [Hy] was calculated using plasma TTR values and the following equation [14]:

$$Hy=Ra\;Tyr\times \frac{\left[{}^{2}H_{4}\right]Tyr\;TTR]}{\left[{}^{2}H_{5}\right]Phe\;TTR}$$

where: [Ra Tyr] is the rate of appearance of tyrosine in plasma (in μmol x kg LBM^−1^ x min^−1^), and [^2^H_4_]Tyr (D_4_-Tyr) TTR and [^2^H_5_] Phe (D_5_-Phe) TTR are the TTR values of [^2^H_4_]Tyr and [^2^H_5_] Phe in plasma, respectively. The rate of phenylalanine disposal to protein synthesis (PS) was calculated by subtracting Hy from Phe Ra.

The kinetics data were expressed also as the fractional conversion rate of phenylalanine disposal, to either hydroxylation or PS.

All data were expressed as means ± standard Error (SE). The one-way Analysis of Variance (ANOVA) and post hoc tests, were employed to compare the data among the three groups. Linear regression analysis to test possible correlations between continuous variables, was employed. The possible confounding effects on some kinetics parameters of age, body weight, body mass index (BMI) and lean body mass (LBM) were analyzed using ANCOVA. The Statistica® Software, version 10, was employed. A p value ≤0.05 was considered statistically significant.

## Results

### Anthropometric, clinical and biochemical data of the study subjects

Age was not different among groups. However, body weight, body mass index (BMI), and estimated lean body mass (LBM, by Hume’s formula) [18], were higher (p<0.05) in the diabetic subjects than in the control group (Table 1). The mean duration of diabetes was longer (p<0.015) in patients with albuminuria [AER+] than in those without [AER-]. Irrespectively of albuminuria, T2DM subjects had increased (p<0.04) plasma glucose concentrations, glycated hemoglobin levels and urinary albumin excretion rate (Table 1). Among T2DM patients, seven had normal urinary AER (<30 mg/day) [AER-] whereas seven exhibited increased AER (>30 mg/day) [AER+]. The [AER+] patients ranged from micro- (two subjects: 42 and 47 mg x day^-1^ respectively) to macro-albuminuria (five subjects: between 0.36–11.7 g x day^-1^). As compared with [AER-] and controls, T2DM [AER+] subjects had higher (p<0.05) systolic blood pressure, urinary albumin excretion rate, plasma creatinine and fibrinogen concentrations and lower GFR. In the [AER+] group, three subjects had normal kidney function (between 100 and 113 ml/min/1.73 m^2^), three were in stage 3 of chronic renal failure (CRF) (GFR between 37–51 ml x min^-1^ x 1.73 m^2(-1)^) and one in stage 4 (GFR: 21 ml x min^-1^ x 1.73 m^2(-1)^). The [AER+] subjects with the highest AER had also the worst kidney function as estimated by GFR. Plasma lipids, proteins, albumin and amino acids (phenylalanine, tyrosine and leucine) were not different among the three groups.

**Table 1:** Clinical and biochemical characteristics of the male T2DM subjects with [AER+] or without [AER-] albuminuria, and of the male healthy controls.

	T2DM [AER+]	T2DM [AER-]	Controls
N	7	7	11
Age (yrs)	55 ± 3	46 ± 4	42 ± 3
Body weight (kg)	90.1 ^a^ ± 2.7	87.6 ^b^ ± 6.0	74.3 ± 2.2
BMI (kg/m^2^)	29.4 ± 0.9 ^b^	28.6 ± 1.9	25.4 ± 0.7
Estimated LBM (kg)	59.5 ± 1.1 ^a^	58.3 ± 2.1 ^b^	53.3 ± 1.2
Systolic blood pressure (mm Hg)	152 ± 7^c^^,^ ^d^	130 ± 4	123 ± 3
Diastolic blood pressure (mm Hg)	91 ± 4	89 ± 2	83 ± 3
Plasma creatinine (μmol/l)	163 ± 34 ^d^^,^^e^	74 ± 4	90 ± 2
Estimated GFR (ml/min/1.73 m^2^)	67 ± 14 ^d^^,^^e^	131 ± 8 ^c^	104 ± 3
*range*	21–110	99–160	83–121
AER (μg/min)	2403 ± 1156 ^b^	7 ± 2	n.d.
*range*	29–8075	3–16	n.d.
Diabetes duration (yrs)	16 ± 3 ^f^	8 ± 1	/
Glycated Hemoglobin (%)	10.5 ± 0.9	9.9 ± 0.6	<4.5
Fasting plasma glucose (mmol/l)	12.8 ± 2.6^a^^,^ ^b^	9.4 ± 0.8 ^d^	4.8 ± 0.1
Total cholesterol (mmol/l)	5.4 ± 0.4	4.9 ± 0.4	5.6 ± 0.5
HDL Cholesterol (mmol/l)	0.9 ± 0.1	1.0 ± 0.1	1.3 ± 0.2
Triglycerides (mmol/l)	2.4 ± 0.3	1.7 ± 0.3	1.4 ± 0.2
Total plasma protein (mg/100 ml)	69 ± 1	71 ± 2	75 ± 2
Albumin (mg/100 ml)	37 ± 2	45 ± 3	42 ± 1
Fibrinogen (mg/100 ml)	480 ± 68 ^a^	373 ± 29	262 ± 13
Plasma Phenylalanine (μmol/l)	51 ± 3	57 ± 4	60 ± 2
Plasma Tyrosine (μmol/l)	52 ± 5	57 ± 6	60 ± 2
Plasma Leucine (μmol/l)	165 ± 13	169 ± 16	153 ± 10

^a^ p≤0.02, and

^b^ p<0.05, vs. Controls.

^c^ p <0.001, vs. Controls, and

^d^ p<0.005, and

^e^ p<0.001 vs T2DM [AER-].

^f^ p≤0.05, T2DM [AER+] vs. T2DM [AER-].

Abbreviations: BMI: Body Mass Index; LBM: Lean Body Mass; HDL: High Density Lipoprotein; GFR: Glomerular Filtration rate; AER: Albumin Excretion Rate.

### Kinetics data

**Fig 1:**
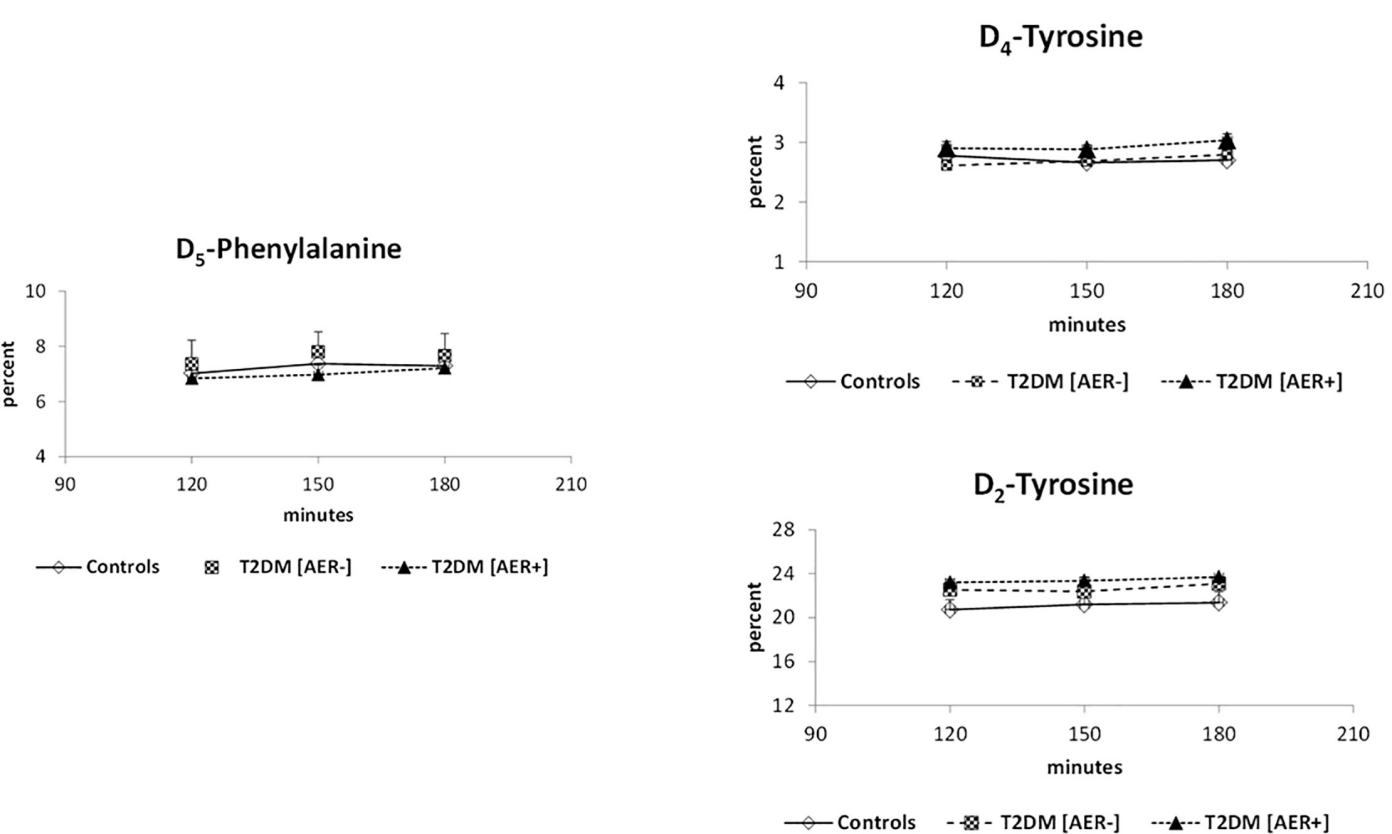
Isotopic steady-state (expressed as isotopic ratio, m/z) of plasma D_5_-phenylalanine (top panel), D_4_-tyrosine (middle panel) and D_2_-tyrosine (bottom panel), in the control group (open symbols and continued lines), the T2DM subjects with normoalbuminuria (T2DM [AER-]) (dotted symbols and dashed lines) and in the T2DM subjects with either micro- or macro-albuminuria (T2DM [AER+] (full symbols and dotted lines). Values are reported as mean ± standard error (SE).

The rates of appearance (Ra) of phenylalanine and tyrosine were not different among groups (Table 2) and their isotopic abundance at steady state is shown in Fig 1.

**Table 2:** Phenylalanine and tyrosine kinetics.

	T2DM [AER+]	T2DM [AER-]	Controls
Phenylalanine Ra	0.93 ± 0.06	0.95 ± 0.07	0.88 ± 0.05
Tyrosine Ra	0.88 ± 0.05	0.95 ± 0.11	0.75 ± 0.09
Phenylalanine to tyrosine	0.21 ± 0.02^a^	0.17 ± 0.02	0.13 ± 0.01
Phenylalanine to PS	0.72 ± 0.06	0.77 ± 0.06	0.75 ± 0.04

The kinetic data were expressed as μmol/kg LBM/min.

^a^ p<0.02 vs. Controls, by ANOVA.

Abbreviations: AER: Albumin Excretion Rate; Ra: rate of appearance; PS: Protein synthesis.

In contrast, in T2DM [AER+] subjects the rate of conversion of phenylalanine to tyrosine (i.e. hydroxylation) was ≈60% greater (p<0.02 by ANOVA and Fisher’s post-hoc test) than that of the control group, whereas it was ≈30% greater, although not significantly (p = 0.095) different than that of controls in T2DM [AER-] (Table 2).

**Fig 2:**
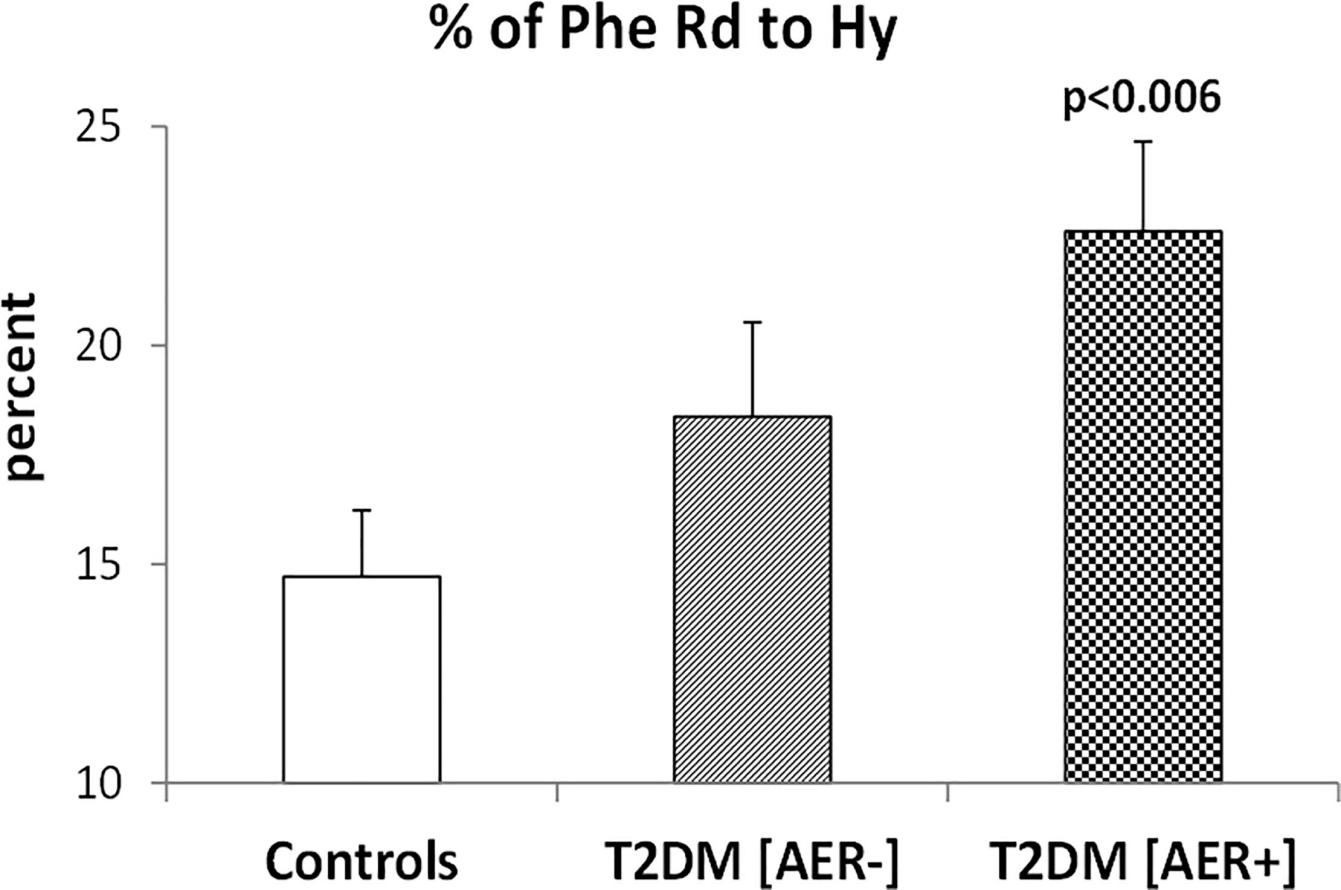
Percent of the rate of phenylalanine Rd used for hydroxylation (Hy), in the control group (left bar), in the T2DM subjects with normoalbuminuria (T2DM [AER-]) (middle bar), and in the in the T2DM subjects with either micro- or macro-albuminuria (T2DM [AER+] (right bar). The p value on top of the bars in the figure denotes the level of significance of the difference of the Controls and of the (T2DM [AER-]) vs. the (T2DM [AER+] (by One-way ANOVA and Fisher’s post hoc tests).

**Fig 3:**
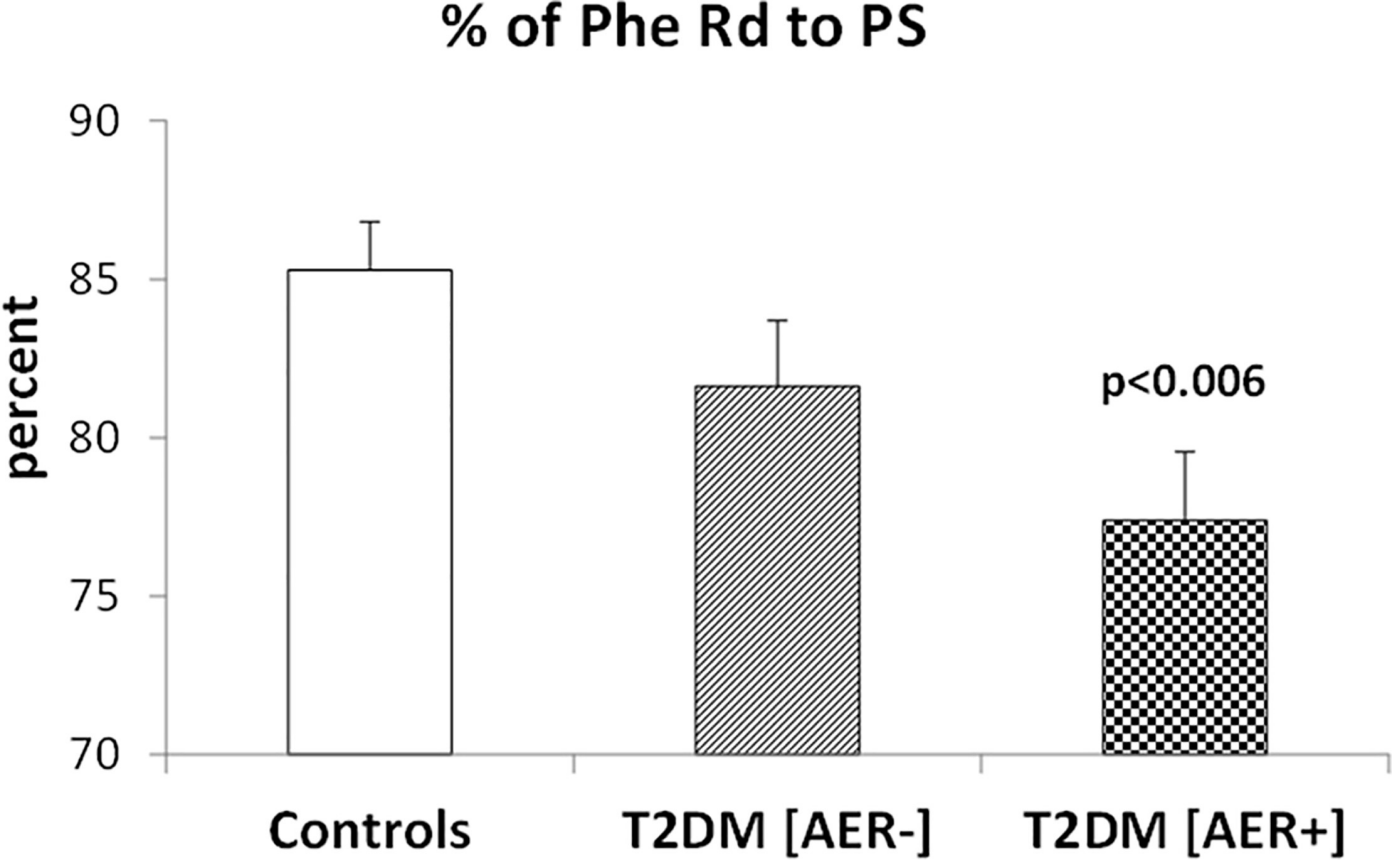
**Percent of the rate of phenylalanine disposal (Rd) (equal to the rate of appearance, Ra, at steady-state) used for protein synthesis (PS), in the control group (left bar), in the T2DM subjects with normoalbuminuria (T2DM [AER-]) (middle bar), and in the in the T2DM subjects with either micro- or macro-albuminuria (T2DM [AER+] (right bar).** The p value on top of the bars in the figure denotes the level of significance of the difference vs. the control group (by One-Way ANOVA and Fisher’s post hoc tests).

In T2DM [AER+] subjects, the fractional conversion rate of phenylalanine disposal to hydroxylation (Hy) (Fig 2) was significantly (p<0.006) greater (by ≈50%) than that of the control group, and ≈25% greater (albeit not significantly, p = 0.17) in the T2DM [AER-] group than in control subjects. Conversely, in T2DM [AER+] group, the fractional Phe disposal to PS (Fig 3) was significantly lower (~10%) than that of the control group, and substantially not different between the T2DM [AER-] group and the controls.

**Fig 4:**
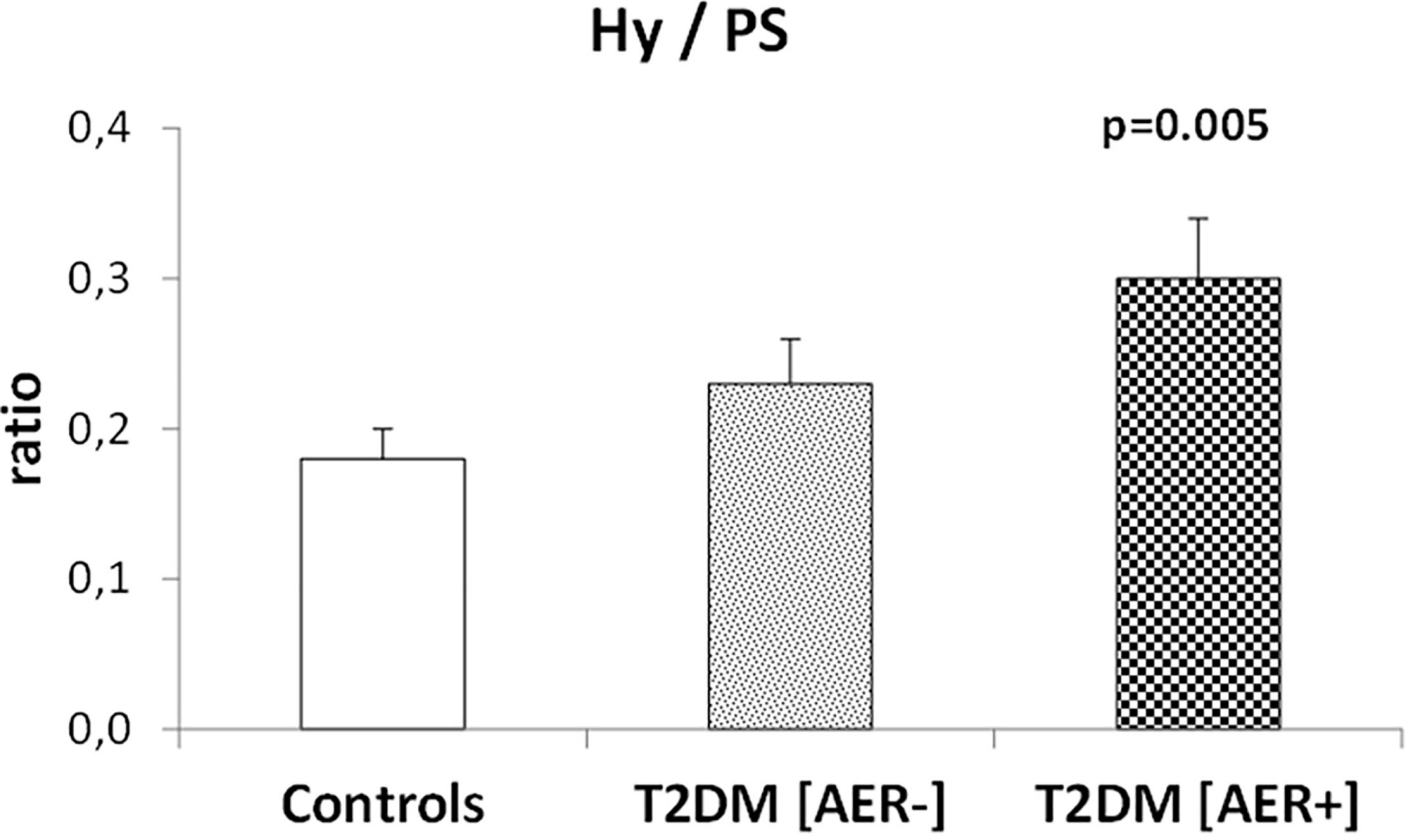
**Ratio between phenylalanine hydroxylation (Hy) and its use for Protein Synthesis (PS), in the control group (left bar), in the T2DM subjects with normoalbuminuria (T2DM [AER-]) (middle bar), and in the in the T2DM subjects with either micro- or macro-albuminuria (T2DM [AER+] (right bar).** The p value on top of the bars in the figure denotes the level of significance of the difference vs. the control group (by One-Way ANOVA and Fisher’s post hoc tests).

As a consequence, in T2DM [AER+] subjects, the ratio between fractional Phe disposal to hydroxylation and to PS was significantly greater (by ≈70%) than that in healthy controls, whereas in T2DM [AER-] patients this ratio was ≈30% greater although not significantly (p = 0.19) than that of the control group (Fig 4).

Since body weight, BMI and LBM were higher (Table 1) in diabetic patients than in control subjects we tested whether these parameters were correlated with phenylalanine and tyrosine kinetics data. No effect by age, body weight, BMI and LBM, alone or in combination, on any of the calculated kinetic parameters was detected by ANCOVA. However, using simple linear regression analysis, significant correlations (p<0.05) were found between phenylalanine hydroxylation and body weight, BMI and LBM, as well as between fractional phenylalanine hydroxylation, use for protein synthesis and the hydroxylation/protein synthesis ratio (HY/PS), and BMI.

## Discussion

In this study, we show that in male T2DM subjects with albuminuria, the partition of phenylalanine disposal between either a catabolic route (i.e. hydroxylation, an irreversible metabolic step) or an anabolic one (protein synthesis), is unbalanced in favor of the former when compared to healthy controls. In T2DM [AER+] patients, phenylalanine hydroxylation expressed both as absolute values and as fraction of total phenylalanine disposal is higher than that of non diabetic controls (Fig 2). Conversely, in T2DM [AER+] the fraction of total phenylalanine disposal to protein synthesis is lower than that of control subjects (Fig 3). As a result, in T2DM [AER+] the ratio between fractional phenylalanine hydroxylation over its use for protein synthesis is significantly greater (by ~70%) than that of control subjects, indicating activation of protein catabolism. Thus, T2DM [AER+] subjects exhibit the most consistent abnormalities in protein turnover, whereas T2DM [AER-] show moderate intermediate albeit not significant changes as compared to those of control subjects (Figs 2–4).

At variance with the increased rates of phenylalanine catabolism, whole-body protein degradation rate, reflected by phenylalanine Ra, was unaltered in the T2DM subjects with albuminuria, in agreement with previous results obtained with leucine tracers in our laboratory [9]. This finding holds true also in populations of T2DM subjects not specifically characterized for the degree of albuminuria [19–21]. Furthermore, in non diabetic patients with proteinuria in the nephrotic range, leucine turnover and oxidation did not differ from those of healthy controls, and were (normally) suppressed by protein restriction [22, 23]. Nevertheless, the investigation of more specific metabolic pathways, such as those measured in this study, may reveal abnormalities not readily apparent just from measurements of whole body flux rates of an essential amino acid. Notably, we have previously reported that both albumin and fibrinogen fractional synthesis rates are greater in T2DM [AER+] than in T2DM [AER-] subjects [9], thus revealing selected abnormalities in body protein turnover not evident at the whole body level.

An issue relevant to the data here presented, is the site of phenylalanine hydroxylation in the human body. Phenylalanine hydroxylation in humans takes place almost exclusively in the kidneys and within the splanchnic area (i.e. the liver) [24, 25]. The kidneys, despite their lower mass, apparently account for the major portion of body phenylalanine hydroxylation, i.e. between 60–63% of total, as directly determined by us and others using a combination of organ catheterization and isotope infusions [24, 25]. In agreement with these premises, in advanced chronic renal failure, whole body phenylalanine hydroxylation was found to be decreased [26–28], likely because of decreased renal hydroxylation. These findings should be discussed in the light of the results here reported. In our study, the degree of renal impairment ranged from 0 (3 patients) to 4 (one patient). However, globally the [AER+] T2DM subjects showed increased (i.e. not *decreased*) phenylalanine hydroxylation, apparently in contrast with the role of the kidney in the hydroxylation reaction. A possible explanation for these apparently contrasting findings could be the degree of renal failure (mild to moderate vs. advanced as in the above-cited previous reports) [26–28]. In another study, the oxidation of ^14^C-phenylalanine given orally was increased in hemodialysis patients as compared to healthy controls [29], suggesting an increased catabolism of this essential amino acid. Although phenylalanine oxidation (i.e. decarboxylation) is a step beyond its hydroxylation to tyrosine [30] these finding nevertheless indicate increased phenylalanine irreversible catabolism.

An alternative explanation is that phenylalanine hydroxylation was actually increased in the other important body site of hydroxylation, i.e. in the splanchnic (liver) district. The splanchnic area extracts most of the orally-administered phenylalanine both in normal [13] and in type 2 diabetic subjects [19]. Using mass balance studies across the splanchnic area and the kidney in human subjects with either normal or decreased renal function, and with measurements of net uptake and release of phenylalanine and tyrosine [31, 32], both CRF and normal subjects exhibited a net uptake of both phenylalanine and tyrosine by the splanchnic area. However, following the ingestion of an amino acid meal, in both groups a release of phenylalanine from the splanchnic bed was observed, that was however greater in CRF than in normal controls [32], and likely reflected an impaired phenylalanine hydroxylation too [26] in agreement with a decreased phenylalanine splanchnic extraction in the former group. If these data are combined with those reporting an increased oxidation of orally-administered phenylalanine in CRF patients [22], it could be suggested that, in CRF, less phenylalanine is available to sustain liver protein synthesis (either of structural or of export proteins). As proof of concept, the synthesis of VLDL-Apo B 100, a liver export protein, is reduced in T2DM subjects with albuminuria and with moderate renal failure [10]. In contrast, an accelerated albumin and fibrinogen synthesis has been reported in T2DM with albuminuria [9] suggesting that the synthesis of individual hepatic proteins may be differently regulated in T2DM with albuminuria and/or mild renal impairment. Further studies are required to elucidate these complex issues.

The role of renal function *per se* should also be addressed. Advanced chronic renal failure (stages 4–5) has been associated with either unchanged [33–36], or decreased [37] whole body protein degradation. Conversely, net protein catabolism, as shown by leucine oxidation, was either increased [33], normal [34] or decreased [35–37]. Although a direct comparison between the present and previous findings is difficult due to the wide variability of the stage of kidney function in our albuminuric patients, results showing normal whole body protein degradation and increased net protein catabolism in advanced CRF fit well with our data based on phenylalanine and tyrosine kinetics.

Although there was a trend in the [AER-] T2DM group towards increased Phe hydroxylation and reduced Phe disposal to protein synthesis, these differences did not attain statistical significance. Therefore, from our data we cannot rule out whether the increased fractional phenylalanine hydroxylation in T2DM is due to coexisting albuminuria, and/or to an interaction between the latter and T2DM. Unfortunately, at the time of the study it was not possible to investigate an additional group of non diabetic subjects with albuminuria. Nevertheless, the intermediate results observed between the T2DM subjects with either normal or increased albuminuria may offer a partial response. In addition, although diabetes duration was longer in [AER+] than in [AER-] T2DM group, the catabolic pattern (i.e. the ratio between hydroxylation and protein synthesis) was not correlated with the duration of the disease (r = 0.28, p = 0.8) suggesting that albuminuria *per se* is the major drive of increased protein catabolism.

In its progression chronic kidney failure is associated with profound changes in protein metabolism, resulting in a catabolic pattern which ultimately determines protein-energy wasting and loss of muscle mass. Although our experiments were performed in the post-absorptive state and therefore the response of phenylalanine kinetics following meal ingestion and/or intravenous nutrition is not readily extrapolated from the current data, a previous study has shown that, in nephrotic and in control subjects consuming either a normal or a low-protein diet, nitrogen balance was maintained equally positive. This situation is the result of an adequate suppression of whole-body protein degradation and of leucine oxidation, and of the simultaneous stimulation of protein synthesis during feeding in either group [22]. Thus, body tissues can apparently activate compensatory mechanisms to minimize protein loss also under conditions of dietary protein restriction. Whether such mechanism(s) involve also changes in phenylalanine hydroxylation needs to be determined.

In [AER+] T2DM patients, body weight and calculated lean body mass were not significantly different from those of control and [AER-] T2DM subjects although they tended to have lower albumin and higher BMI. Therefore, the observed changes of whole body phenylalanine (and protein) kinetics could reflect early derangements in protein metabolism, possibly contributing to the development of sarcopenia as the disease progresses.

Although the diabetic subjects had greater body weight, BMI and LBM than the control group (Table 1), no confounding effect of these variables, on phenylalanine hydroxylation (HY), fractional phenylalanine utilization for protein synthesis and HY/PS ratio was detected by ANCOVA. However, simple regression analysis showed direct correlations between these kinetic variables, and body weight, BMI and LBM. Thus, it cannot be excluded that these anthropometric variables had some direct effect on phenylalanine catabolism. Further studied with strictly-matched control and diabetic subjects are warranted.

Albuminuria is considered a negative marker for morbidity and mortality in the general population [38, 39] as well as in specific conditions such as T2DM [3, 40]. As regards hypertension, it is usually present in about 70% of T2DM subjects [41]. Although hypertension *per se* can be associated to increased urinary albumin excretion, hypertension is also a frequent feature of diabetic nephropathy, therefore a clear-cut distinction between the two conditions as the primary cause for albuminuria cannot be made.

In conclusion, in this study we show that T2DM subjects with albuminuria exhibit features compatible with a catabolic protein status, as reflected by increased irreversible catabolism of the essential amino acid phenylalanine that could anticipate future alterations in nutritional and metabolic parameters.
